# Synergistic Impact of Sleep Disturbance and Social Isolation in Adult but Not Adolescent Female Mice

**DOI:** 10.1111/jsr.70213

**Published:** 2025-09-25

**Authors:** Christine Egebjerg, Birgitte Rahbek Kornum

**Affiliations:** ^1^ Department of Neuroscience University of Copenhagen Copenhagen Denmark

**Keywords:** anxiety, depression, home‐cage behaviour, neurodevelopment, sleep

## Abstract

Sleep quality and social isolation are two of the numerous environmental, social and health‐related factors that contribute to major depressive disorder (MDD). In human studies, a strong association has been found between sleep quality and perceived loneliness, with each potentially exacerbating the other. In mouse studies, sleep deprivation is performed on either group‐housed or individually housed mice, depending on the protocol, but the effect of social isolation is often not assessed. Here, we aimed to investigate the potential synergistic effects of sleep disturbances and social isolation in adolescent (Postnatal day 36) and young adult (9 weeks old) female mice. The mice were subjected to sleep disturbances for 7 days, consisting of 4 h of sleep restriction during the light phase, while group‐ or single‐housed for 7 days. Both the individual and combined effects of sleep disturbance and social isolation were assessed. Our findings reveal significantly longer immobility in the tail suspension test in young adult mice after 7 days of sleep disturbance + social isolation compared to those in the sleep disturbance + socially housed group. The same effect was not seen with adolescent mice. This interaction between sleep disturbance and social isolation in the young adult group suggests a synergistic effect. In conclusion, single housing of mice can change the behavioural outcome of a sleep disturbance protocol. Further, adolescent mice appeared more resilient to the adverse behavioural effects of sleep disturbance in combination with social isolation than young adult mice.

## 1 Introduction

Sleep is vital for the human body and mind, yet many people struggle to achieve enough good‐quality sleep. Poor sleep can lead to unwanted consequences, including impaired memory, emotional dysregulation and reduced attention, all of which are crucial for a healthy functioning brain (Medic et al. 2017). Sleep disturbances (SDs) are also closely linked to various mental health disorders, including major depressive disorder (MDD) (Mendlewicz 2009).

To understand the consequences of SDs on mental health, rodent studies are often performed in which a sleep deprivation protocol is performed, followed by an evaluation of molecular, cellular, or behavioural endpoint measurements. There are many approaches to sleep deprivation in rodents, spanning from the introduction of novel objects to the cage (Egebjerg et al. 2025) or gentle handling to closed‐loop optogenetic stimulation (Rolls et al. 2011). Some of these protocols can be performed on group‐housed mice, while other protocols require single housing of the animal. It is well known that social isolation (SI) affects rodents and can affect their physiology (Späni et al. 2003), but often this variable is not considered in studies of the behavioural or neurophysiological response to sleep deprivation.

This is surprising given the high relevance of SI as a risk factor for mental health disorders. Depression, as an example, is a very heterogeneous disease, and multiple factors can influence the risk of developing it, not only SD (Schaakxs et al. 2017). SI and loneliness are amongst the factors associated with depression (van Winkel et al. 2017). Importantly, one study investigated the envirome (total set of environmental factors, both present and past, that affect the state, and in particular the disease state, of an organism) of MDD and concluded that environmental, lifestyle, social and other health‐related factors are interconnected, work synergistically and must be evaluated together to understand MDD (Hullam et al. 2019). This means that even though studies of sleep deprivation always include a non‐sleep deprived control group, the housing condition could work in synergy with the lack of sleep and influence the outcome of the study. Indeed, using EEG measurements, it has been shown that mice respond differently to sleep deprivation depending on whether they are socially isolated or pair‐housed. Five‐week SI of adult male mice resulted in a blunted delta power increase during recovery sleep in the late dark phase compared to the paired controls after sleep deprivation (Kaushal et al. 2012).

In humans, sleep quality has also been investigated in relation to SI and loneliness. A study in young adults investigated the association between perceived loneliness (UCLA Loneliness Scale scores) and sleep quality (the Pittsburgh Sleep Quality Index) and reported a strong association between the scores. They concluded that individuals who reported perceived loneliness had decreased sleep quality (Matthews et al. 2017). A systematic review including 27 articles suggested that the link between sleep and perceived loneliness is complex. Self‐reported sleep quality decreased with increased perceived loneliness, but no association with the duration of sleep has been found (Griffin et al. 2020). Specific measures of sleep such as sleep adequacy, sleep satisfaction and change in sleep also had a bivariate association with perceived loneliness (Griffin et al. 2020).

Although often confused, SI and perceived loneliness are two distinct psychosocial principles (Taylor 2020). Perceived loneliness refers to the feeling of being lonely, whereas SI means having no contact with others (Taylor et al. 2023). Both can occur independently of each other. One study investigated the association between loneliness, SI and depression in young adults. The study found that there was an association between perceived loneliness and depressive symptoms (Matthews et al. 2016). SI and depressive symptoms were not associated, suggesting that isolation without perceived loneliness is not adverse to human health (Matthews et al. 2016).

Previously, it was almost impossible to study SI, but the COVID‐19 quarantine created a situation of forced SI. Multiple sleep studies reported changes in sleep, such as lower‐quality sleep (Franceschini et al. 2020), insomnia (Morin et al. 2021), delays in sleep timing and increased time in bed (Yuan et al. 2022) during the COVID‐19 quarantines. However, it is essential to note that the COVID‐19 quarantine also affected mental health on other parameters, such as financial uncertainty (Godinić and Obrenovic 2020), increased worrying (Samuels et al. 2021) and fear of infection (Şimşir et al. 2022). The diversity of sleep changes shows how differently people reacted to the COVID‐19 quarantine and how difficult it is to measure the impact of SI on humans.

Rodent models have the advantage that they allow for studies of single factors taken from the complexity of the human situation. Multiple studies have been performed on long‐term isolated mice demonstrating their impact. Post‐weaning SI has been intensively studied and has shown increased aggression, anxiety‐ and depression‐like behaviours (Huang et al. 2017; Takahashi 2025; Toth et al. 2011). These behaviours have also been shown in adult mice (Guo et al. 2004; Takahashi 2025). In sleep deprivation studies, the possible SI is often short and therefore possibly less impactful, but this has to our knowledge never been systematically studied.

In this study, we aim to investigate the synergistic effects of SDs and SI by using home‐cage activity, tail suspension test (TST) and open field test (OFT) to assess behavioural changes. We used only female mice due to the high prevalence of mood disorders in women (Bromet et al. 2011; Mong and Cusmano 2016; Zhang and Wing 2006). Here, we show that the housing condition of the mice indeed does alter the behavioural response to SD. To determine age‐specific effects, we included adolescent and young adult female mice. We hypothesise that adolescent mice would be particularly vulnerable to SD and SI in combination compared to adults, but this hypothesis was not confirmed.

## 2 Methods

### 2.1 Animals

C57BL/6JTac female mice from Taconic were used for these experiments. All mice used in the experiments were kept on a 12‐h light–dark cycle with access to ad libitum food and water at constant temperature and humidity. All protocols were approved by the Danish Animal Experimental Inspectorate (licence number: 2022‐15‐0201‐01194), and the experimental procedures were conducted following the Directive 2010/63/EU of the European Parliament and Council on the protection of animals used for scientific purposes.

### 2.2 Experimental Setup

Two age groups were used: adolescent mice (Postnatal Day [p]36) and young adult mice (9 weeks old). Within each age group, mice were divided into four experimental groups: control (Ctrl + social), sleep disturbance (SD + social), social isolation (Ctrl + SI) and both SD + SI. SD was induced using novel objects as previously validated (Egebjerg et al. 2025) for 4 h starting at zeitgeber time (ZT)2 for 7 consecutive days. We started at ZT2 to minimise the jetlag effect from SD. Egebjerg et al. used EEG/EMG to validate the SD method and concluded that the mice were sleep‐restricted during exposure to novel objects but would catch up on their sleep after the intervention. This indicates that the mice are sleep‐disturbed and only deprived during exposure to objects (Egebjerg et al. 2025). We expect the mice in this study to behave similarly to Egebjerg et al. 2025, since the setup was performed in an identical manner (same objects, same animal house etc.).

**FIGURE 1:**
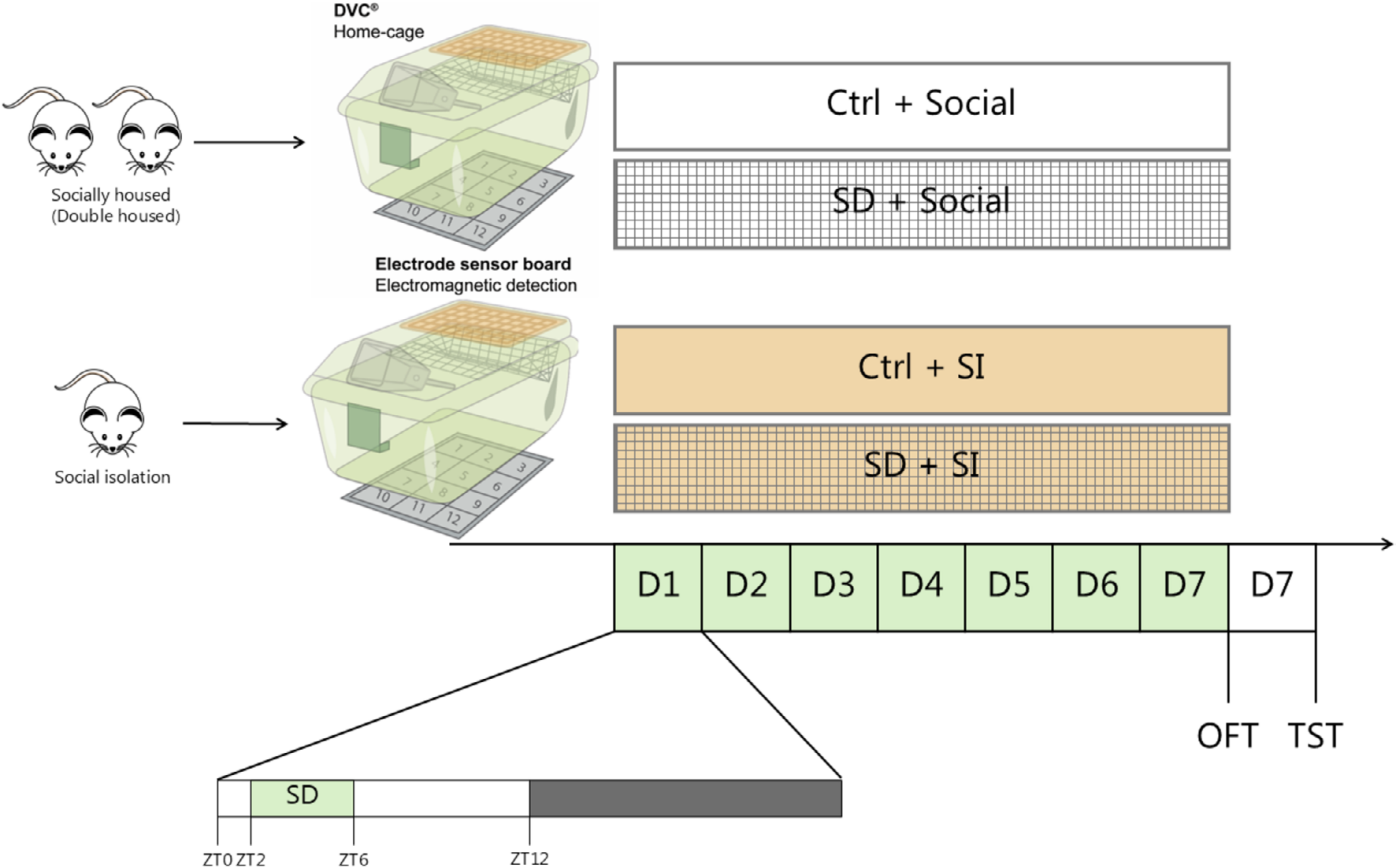
Experimental overview. The study included adolescent (postnatal day (p)36) and young adult (9 weeks old) mice, divided into four groups: Control and socially housed (Ctrl + social), sleep‐disturbed and socially housed (SD + social), control and socially isolated (Ctrl + SI) and both sleep‐disturbed and isolated (SD + SI). Before the start of the experiment, the mice were placed in pairs or in SI in the DVC home‐cages to track their activity during SI and SD. SD was performed with novel objects for 4 h daily at zeitgeber time (ZT)2–4 for 7 consecutive days. On the last day of SD, the mice were tested in an open field test (OFT) and a tail suspension test (TST) the day after. All behavioural tests were performed between ZT8–12.

At the start of the experiment, all mice were placed in new cages, and the Ctrl+SI and SD + SI groups were socially isolated at the start of the experiment (Figure 1). All sleep‐disturbed mice (including SD + SI) were provided with objects carrying odours from other same‐sex animals. When given the novel object, the lid of the cage was put back to leave the mice as undisturbed as possible. The mice could potentially see other mice in the housing rack, and during the SD intervention, the cages were placed with a similar distance between them. All cages included in the setup, regardless of housing condition, were transferred to the same experimental room during the SD intervention.

For home‐cage activity tracking, data were accumulated from adolescent mice housed in 25 Ctrl + social and 14 SD + social cages, as well as 10 Ctrl + SI and six SD + SI cages. For young adult mice, data were collected from 4 cages each for Ctrl + social and SD + social and 8 cages each for Ctrl + SI and SD + SI. The results of the TST and the OFT from the adolescent Social + SD have previously been published in another context, as well as one cohort of adolescent Social + Ctrl (Figure S1, Experimental Group 2) (Egebjerg et al. 2024). In the behavioural experiments, we used 26 adolescent Ctrl + social, 8 adolescent SD + social, 10 adolescent Ctrl + SI and 7 adolescent SD + SI mice. For young adults, we included eight mice per group across all 4 conditions.

Due to experimental limitations, the data of the adolescent cohort was gathered by repeating the experiment multiple times. Light, temperature and box placement were exactly the same. To ensure no changes between the behavioural test rounds, a control group was present in each setup to indicate possible changes from time to time, resulting in an overall larger control group. The control groups from each round of experiments were compared, and no significant differences were found (Figure S1). Furthermore, all data analysis was repeated by downscaling the Social + Ctrl and Social + SD to *n* = 10. This was done by random selection of data points, ensuring that each round was represented. Significant differences remained significant after downscaling (Figures S2–S4).

### 2.3 Home‐Cage Activity Tracking—Digital Ventilated Cages (DVCs)

Mice were housed in specialised DVC (Techniplast, DGM500, Buguggiate, Italy) to record home‐cage activity for each cage during the intervention. Below the cages, a board of 12 electrodes measures the total locomotion activity by using electrical capacitance technology (Iannello 2019). The animal locomotion index is determined by the number of electrodes that detect activity. A score of 0% means no electrodes detected activity, while a score of 100% indicates that all 12 electrodes are active simultaneously. The mice were habituated to the DVC cages for 7 days before measuring. The first 6 days of intervention were recorded in the DVC cages, but on the seventh day, the mice were kept in the same room where the SD was performed. This was done to minimise the change in the environment before the behavioural tests.

The DVC cages sample the total activity of the cage. Therefore, we chose to divide the locomotion activity for the double‐housed animals so that the datapoints would represent one animal, making it easier to compare them to the single‐housed animals. This means that the data points for the double‐housed animals do not represent the precise individual activity but rather a mean of the activity. However, we would expect the mice to move approximately the same, as they are going through an identical SD paradigm.

### 2.4 Behavioural Tests

**FIGURE 2:**
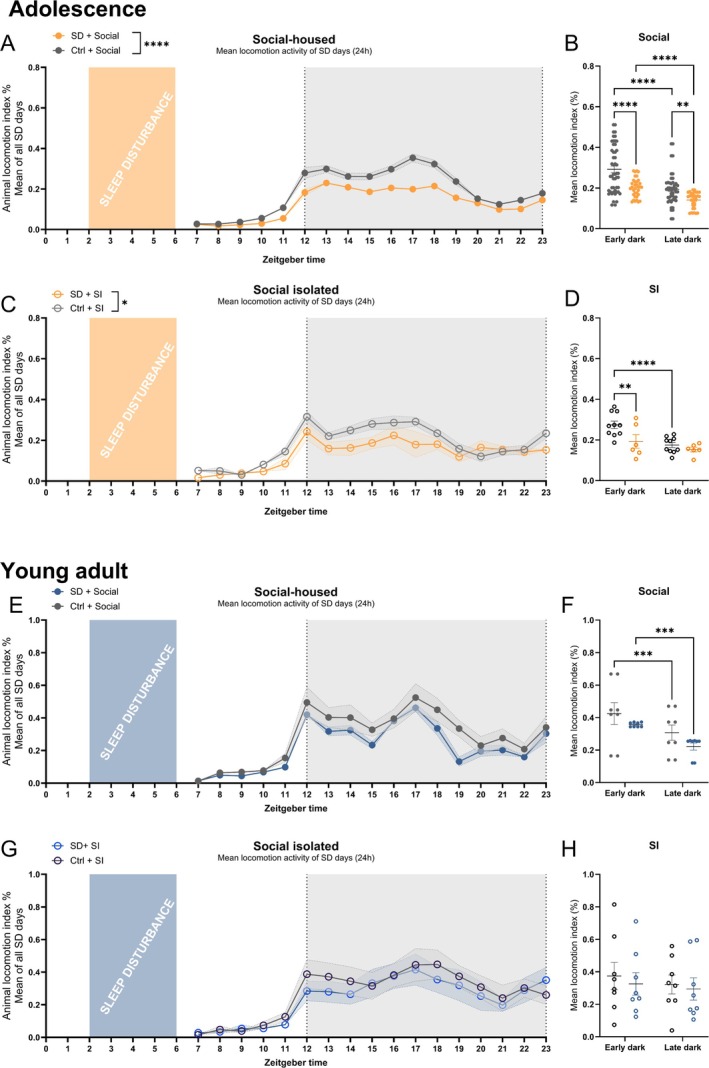
Adolescent mice have decreased home‐cage activity after sleep disturbance (SD), but not the adults. Adolescent (p36) and adult (9 weeks) mice were either sleep‐disturbed (SD) or left undisturbed (Ctrl), and housed either socially (group‐housed) or in social isolation (SI). (A, C, E and G) Mean locomotion activity index over 6 days/h plotted from zeitgeber time (ZT)7. (A) Socially housed adolescent mice (SD vs. Ctrl), (*n* = 28–50). (B) Data as in (A), separated into early (ZT12–17) and late dark phases (ZT18–23) (*n* = 28–50). (C) Socially isolated adolescent mice (SD vs. Ctrl) (*n* = 6–10). (D) Data as in (C), separated into early and late dark phases (*n* = 5–10). (E) Socially housed young adult mice (SD vs. Ctrl) (*n* = 8). (F) Data as in (E), separated into early and late dark phases (*n* = 8). (G) Socially isolated young adult mice (SD vs. Ctrl) (*n* = 8). (H) Data as in (G), separated into early and late dark phases (*n* = 8). All data represented were analysed using a repeated‐measures two‐way ANOVA and followed by an uncorrected Fisher's LSD post hoc test for phase comparisons (Table S1). *p < 0.05, **p < 0.01, ***p < 0.001, ****p < 0.0001. All bar plots show mean ± SEM. Statistics found in Table S1.

All mice were moved to the behavioural experimental room 1 h before the start of the test to habituate to the room. The experimenter left the room during testing. Both the OFT and the TST were performed in the timespan of ZT8–12. Results of TST and OFT of the SD (Figures 2A and 4A–C) and controls of the second repetition (Figures S1, 2A and 4C) from the adolescence cohort have previously been published in (Egebjerg et al. 2024).

### 2.5 Open Field Test (OFT)

Each mouse was placed carefully in an acrylic box (*L*: 37 cm × *W*: 21 cm × *H*: 19 cm) for 10 min of video recording. The boxes were cleaned with a 70% ethanol solution before the experimental start and between each trial to ensure clean cages and the same exposure to the smell of ethanol. Ethovision was used to quantify distance travelled, velocity, and distances to the centre point. In the OFT, the anxiety‐like behaviour is primarily inferred from centrophobic behaviour. The mouse will stay close to the walls and further away from the centre if it shows anxiety‐like behaviours. For this reason, we used the distance to the centre as a readout because we use a rectangular box rather than a square or a circle, as used in most setups. The sampling rate of the distance to the centre point was 1 s, and the data was plotted against the 10‐min recording to calculate the area under the curve. This measure was calculated for each mouse and used to determine how close the mouse was to the edge of the arena.

### 2.6 Tail Suspension Test (TST)

The mice were suspended by their tails with adhered tape for 6 min in an acrylic box with a hock 30 cm from the table. The tape was placed approximately 1.5 cm from the tail tip. Immobility was determined by manually scoring using Ethovision software in a blinded manner. Immobility was determined following a published protocol (Can et al. 2012).

## 3 Statistics

Statistical analyses and data visualisation were conducted using GraphPad Prism v10.2.3 software (San Diego, CA, USA). All presented data are expressed as mean ± standard error of the mean. All data were assumed to follow a Gaussian distribution. A two‐way ANOVA with repeated measures was used to analyse the home‐cage activity. Activity in the early and late dark phases was analysed by a repeated‐measures ANOVA with an uncorrected Fisher's LSD. A three‐way ANOVA and a Tukey multiple comparisons test were used to interpret the effects of the three independent factors age, housing condition (social vs. SI) and sleep disturbance (Ctrl vs. SD) in the total immobility of TST and the measures of OFT. The TST data over time were analysed using a three‐way ANOVA with repeated measures considering the factors of time, housing condition (social vs. SI) and sleep disruption (Ctrl vs. SD). All *F* and *p* values from the three‐way ANOVA are reported in Tables S1–S3. Significant ANOVAs are indicated by *F* test statistics and *p* values, with *p* < 0.05 considered statistically significant, denoted in figures as *p < 0.05, **p < 0.01, ***p < 0.001.

## 4 Results

### 4.1 Adolescent Mice Decrease Locomotion Activity After SD, but Young Adults Do Not

We measured the total locomotion activity in each cage using the DVC system to elucidate the effects of SD and SI over time. The locomotion activity data was extracted after SD for the first 6 days of SD. On the seventh day, no data is available, as the behavioural tests were performed in the late light phase on the seventh day. The control groups experiencing no SD and no SI (Ctrl + social) show the expected activity pattern for C57BL/6 mice with the highest activity levels in the early dark phase, and this pattern was present irrespective of age (Figure 2A,E). The home‐cage activity was lower in the SD both for the social and SI housed adolescent mice (Figure 2A, *F*(1, 82) = 24.25, *p* < 0.0001, and Figure 2C, *F*(1, 14) = 6.046, *p* < 0.05), whereas there are no changes in locomotion activity of the young adult groups (Figure 2E,G). This indicates that there is an age‐depending decrease in home‐cage activity in response to SD.

To further explore the activity dynamics, we compared activity during the early (ZT12–17) and late (ZT18–23) dark phases. The normal pattern of more locomotor activity in the early dark phase compared to the late dark phase was maintained in group‐housed adolescents in the presence of SD (Figure 2B, *p* < 0.001) or in the presence of SI (Figure 2D, *p* < 0.0001), but was lost in the SD + SI group (Figure 2D, *p* > 0.05). In adult mice, this pattern was maintained in the group‐housed mice but was disrupted in both SI groups (Figure 2H).

### 4.2 Young Adult Mice Exhibit More Despair‐Like Behaviour When Exposed to Both SD and SI Than SD Alone

Our next objective was to investigate whether SD or SI independently led to despair‐like behaviour and whether their combination might produce a synergistic effect.

**FIGURE 3:**
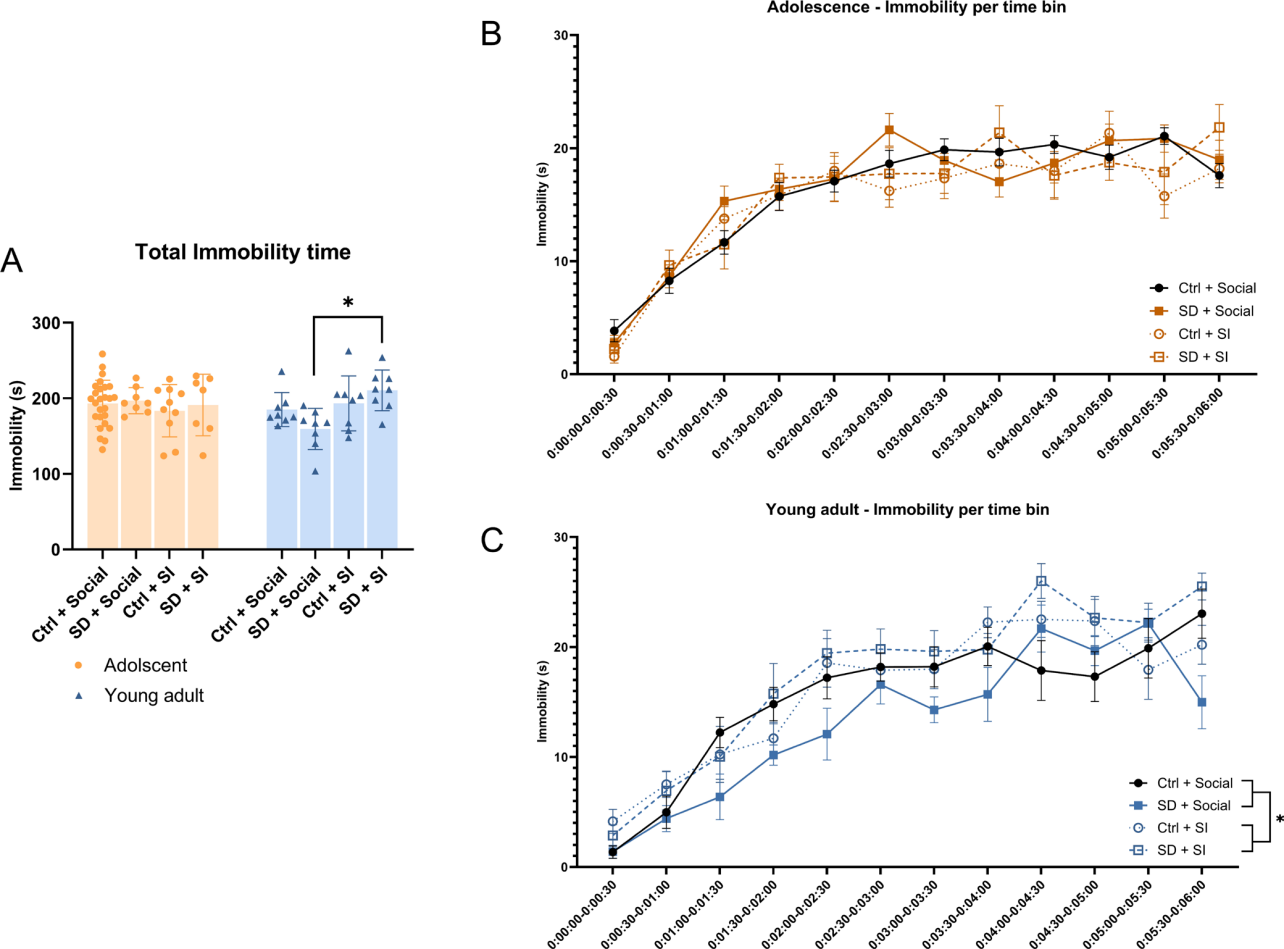
Adult mice had increased immobility time after sleep disturbance and social isolation (SD + SI) compared to SD group‐housed mice. Adolescent (p36) and adult (9w) mice were sleep‐disturbed (SD) or left undisrupted (Ctrl), social‐isolated (SI) or group‐housed (social). A tail suspension test (TST) was performed to elucidate despair‐like behaviour. (A) Total immobility time for all adolescent groups (*n* = 7–26) and young adult mice (*n* = 8). A three‐way ANOVA and post hoc Tukey multiple comparisons test, *p < 0.05. (B) TST immobility over the 6‐min test duration in the adolescent group (*n* = 7–26). A three‐way ANOVA with repeated measures was used. (C) TST immobility over the six‐minute test duration in the young adult group (*n* = 8). A three‐way ANOVA with repeated measures, *p < 0.05. All data are plotted with mean ± standard error of the mean. Statistics found in Tables S2 and S3.

In the young adult group, increased immobility in the TST was seen when SD was combined with SI compared to SD alone (Figure 3A, *p* < 0.01). No significant changes were observed between any of the other adult experimental groups. In adolescent mice, we found no alterations in despair‐like behaviour across all groups (Figure 3A). There was an interaction between age and whether the mice were socially housed or in SI (Table S2, *p* < 0.05), which indicates that young adult mice are more sensitive to being isolated for one week compared to adolescent mice.

The six‐minute TST period was segmented into 30‐s intervals to track changes over time. Also, with this analysis, no significant differences in immobility were observed across the adolescent groups. We did see an increased immobility with time, as expected (Figure 3B, Table S3, *p* < 0.0001). In this analysis, young adults subjected to SI exhibited increased immobility compared to the socially housed mice (Figure 3C, Table S3, *p* < 0.01), and the statistical analysis demonstrated a significant interaction between housing conditions and SD (Figure 3C, Table S3, *p* < 0.05). These results indicate a possible synergistic effect of the combination of SD and SI in young adult female mice.

**FIGURE 4:**
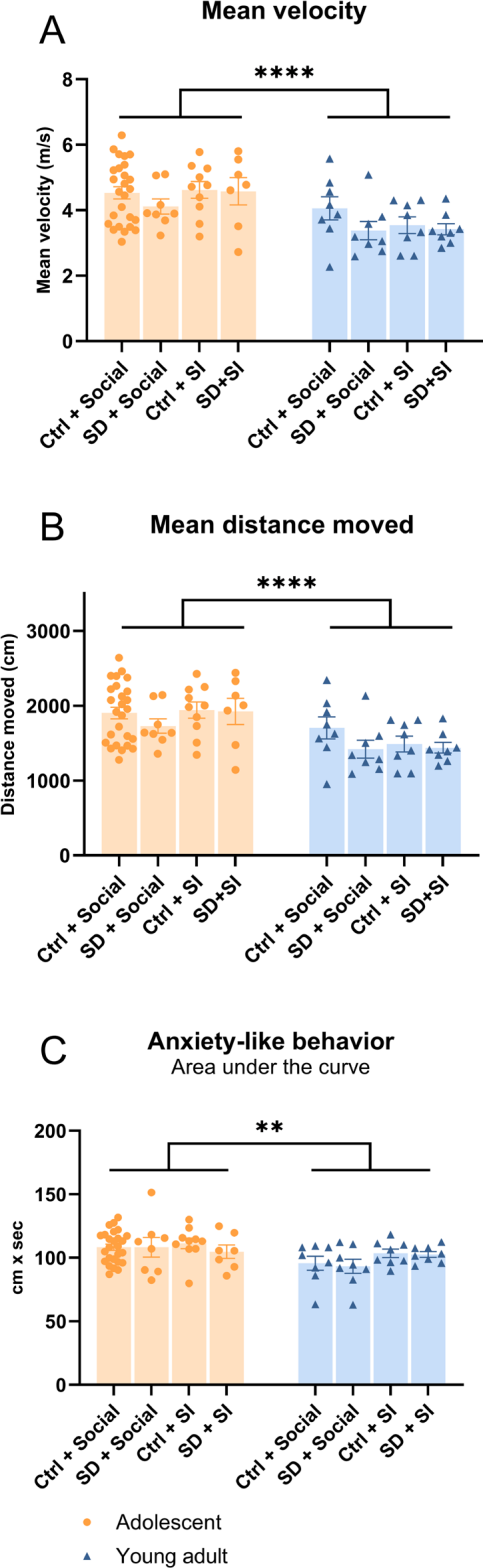
All open field measures (mean velocity, mean distance moved and anxiety‐like behaviour) were significantly decreased in the young adult groups compared to the adolescents. Adolescent (p36) and adult (9w) mice were sleep‐disturbed (SD) or left undisrupted (Ctrl), social‐isolated (SI) or group‐housed (social). An open field test (OFT) was performed to elucidate anxiety‐like behaviour. (A) Mean velocity, (B) mean distance moved and (C) anxiety‐like behaviour were measured for both adolescent (*n* = 7–26) and young adult (*n* = 8) mice. A three‐way ANOVA post hoc Tukey multiple comparisons test was used, **p < 0.01, ****p < 0.0001. All data are plotted with mean ± standard error of the mean. Statistics found in the Table S4.

In the OFT, an overall age difference was observed in velocity (Figure 4A, Table S4, *p* < 0.0001), distance travelled (Figure 4B, Table S4, *p* < 0.0001) and anxiety‐like behaviour (Figure 4C, Table S4, *p* < 0.01). No other changes were observed in the OFT, neither due to SD or SI between or within age groups (Figure 4). Our data show that despair‐like behaviour after SD in adult female mice is affected by housing conditions. The same effect is not seen for anxiety‐like behaviour. For adolescent mice, the housing condition did not affect any readouts.

## 5 Discussion

We here show that in young adult female mice, SD in the context of SI causes a significant increase in despair‐like behaviour compared to SD alone. The same was not the case for adolescent mice, where neither SD, SI nor their combination causes an increase in despair‐ or anxiety‐like behaviour. These results suggest that adolescent mice are less sensitive to the effects of SD and SI than young adult mice. Further, the results show that it is crucial to consider housing conditions when interpreting the effects of sleep deprivation in adult mice.

To assess the efficiency and effect of the SD, we used the home‐cage locomotion activity. We expected the mice experiencing SD to sleep more after the intervention, thus having less home‐cage activity. Only the adolescent mice showed a significant decrease in home‐cage activity after SD, which could indicate a stronger response to the SD compared to the young adult mice. This suggests a differentiated age response to the SD paradigm. However, only the young adult mice showed negative consequences from SD in a behavioural test. Thus, changes in home‐cage activity as a consequence of SD could be a compensatory response, allowing the adolescent mice to cope with SD.

Murack et al. showed that with an 8‐day, 4‐h subchronic SD by gentle handling, adolescent mice had increased immobility in the forced swim test. This was not the case for adult mice (Murack et al. 2021). This indicates that in that setup, the adolescent mice were more vulnerable to SD than the adults, in contrast to our results (Murack et al. 2021). The difference in the SD paradigm could explain the difference, but also the amount of induced sleep loss with the different methods. We used an SD method based on voluntary wakefulness with no elevation of faecal corticosterone (Egebjerg et al. 2025). Murack et al. (2021) used gentle handling and showed that the female mice had elevated serum corticosterone, indicating a more stressful paradigm, possibly resulting in adverse consequences. In general, not only housing conditions but also stress could be a significant confounder in the behavioural consequences previously reported for SD in adolescent mice (Huang et al. 2022; Murack et al. 2021; Xue et al. 2023).

We here take advantage of a low‐stress SD protocol where novel objects induce voluntary wakefulness. This could be considered a short enrichment of the home cage. Environmental enrichment has been demonstrated to mitigate the effects of stress paradigms. Therefore, introducing novel objects to induce SD might interfere with behavioural outcomes (Benaroya‐Milshtein et al. 2004). In addition, one object is a running wheel, which could increase locomotion activity and is also known to decrease the effects of stressful insults (Otsuka et al. 2016). However, a positive effect of enrichment and increased activity could not be detected in our data, suggesting that the effect is minor. It could be interesting to study whether a synergistic effect with SI would also occur for other sleep deprivation protocols.

Multiple studies show that SI of adolescent mice can cause anxiety and depression‐like behaviours (Amiri et al. 2015; Huang et al. 2017; Li et al. 2021). We did not observe such effects after our seven‐day isolation paradigm, showing that it is a mild SI paradigm compared to the previously reported depressive‐like effects using more than two weeks of SI (Amiri et al. 2015; Huang et al. 2017; Li et al. 2021). We chose a shorter SI to avoid masking the effect of the SD and instead investigate more minor insults that may affect each other and cause adverse behaviours. One other study reports similar results to ours regarding SI. Fuhrmann and colleagues investigated responses to 3 weeks of SI in middle‐aged mice (10–11 months) and adolescents (p21). They showed that the middle‐aged groups displayed depression‐ and anxiety‐like behaviour as well as decreased spatial memory after 3 weeks of SI, but the same was not the case for the adolescents (Fuhrmann et al. 2015). This study and our results suggest that adolescent mice could be more resilient to SI and SD compared to an adult cohort.

The adolescent brain is known to have different properties compared to the adult brain. Whether such differences could have a protective effect is unknown. The high plasticity in the adolescent brain could protect the brain from minor insults, whereas the adult brain is fully mature and might be less resilient (Fuhrmann et al. 2015). Neuroplasticity is defined as ‘the ability of the nervous system to change its activity in response to intrinsic or extrinsic stimuli by reorganising its structure, functions, or connections’, which exactly is what is needed to respond to minor insults (Mateos‐Aparicio and Rodríguez‐Moreno 2019). SD has been shown to impair plasticity (Areal et al. 2017) as well as SI (Ieraci et al. 2016), but the adolescent mice's increased plasticity might induce a compensatory response to rescue the behavioural effects.

Another possibility is that adolescent mice deal with the stress induced by OFT and TST differently than young adults. The stressful situation of the behavioural test could hide possible differences between SD and SI in the behavioural outcomes. In the OFT, all parameters had an overall age effect, but there were no differences within the age groups. It has been shown previously that C57BL/6JTac mice increase activity with age in an OFT, which is the opposite of our result (Dixon and Defries 1968). This suggests that young adult mice may exhibit a different behaviour in the OFT. However, since the control group also shows changes with age, the observed differences are not attributable to SD or SI. Advanced home‐cage behavioural analysis could be used to reveal subtle variations and behavioural changes not captured by OFT and TST in the adolescent group. Using advanced home‐cage behaviour as a readout would also minimise the risk of contaminating the behavioural outcomes with a stress response.

We chose to test female mice due to the increased risk of developing MDD in females (Bromet et al. 2011). While the estrous cycle in females may introduce variability in the data, a previous study found that it has minimal effects on mouse sleep patterns (Koehl et al. 2003). Therefore, we do not anticipate a significant impact of the estrous cycle on our experiment. Male and female rodent behaviour can differ, especially related to social behaviours. For example, males exhibit aggressive behaviour towards novel intruders due to territorial aggression (Miczek et al. 2001), whereas female mice do not show aggression towards intruders but can show maternal aggression to defend their pups (Hurst 1987; Miczek et al. 2001). Therefore, male mice are often housed individually compared to females. However, male mice experience even worse adverse effects of SI than female mice, suggesting they might exhibit heightened sensitivity to SI (Guo et al. 2004). A previous study showed similar effects of SD in adolescent mice between sexes (Murack et al. 2021). Therefore, while we do not anticipate that the sex of the mice would drastically alter the results obtained from females, it would be interesting to replicate the study in males to confirm this.

## 6 Conclusion

Altogether, we show that 1 week of SI and SD has a synergistically adverse effect on young adult female mice. The same was not the case for adolescent mice in contrast to what was expected from human studies. Instead, adolescent mice appear more resilient to SI and SD compared to young adult mice.

## Author Contributions

**Birgitte Rahbek Kornum:** conceptualization, funding acquisition, writing – review and editing, project administration, supervision. **Christine Egebjerg:** conceptualization, investigation, writing – original draft, formal analysis, methodology.

## Conflicts of Interest

B.R.K. is a founder and CEO of Ceremedy Aps. The work in this paper is not related to the company. The other author declares no conflicts of interest.

## Supporting information


**Figure S1:** No significant differences between adolescent controls. Controls of the repeated cohorts (first control against social isolation [SI], second control against sleep disturbance [SD] and third control against both SD + SI) were tested to ensure no difference between them. Statistics found in Table S5.
**Figure S2:** Downscaling by random selection from each cohort shows similar results as in the main manuscript. Adolescent (p36) and adult (9 weeks) mice were either sleep‐disturbed (SD) or left undisturbed (Ctrl), and housed either socially (group‐housed) or in social isolation (SI). (A, C, E, G) Mean locomotion activity index over 6 days/h plotted from zeitgeber time (ZT) (A) socially housed adolescent mice (SD vs. Ctrl), 7 (*n* = 10). (B) Data from (A), separated early (ZT12‐17) and late dark phases (ZT18‐23) (*n* = 10). (C) Socially isolated adolescent mice (SD vs. Ctrl) (*n* = 6–10). (D) Data from (C), separated into early and late dark phases (*n* = 5–10). (E) Socially housed young adult mice (SD vs. Ctrl) (*n* = 8). (F) Data from (E), separated into early and late dark phases (*n* = 8). (G) Socially isolated young adult mice (SD vs. Ctrl) (*n* = 8). (H) Data from (G), separated into early and late dark phases (*n* = 8). Data analysed using repeated‐measures two‐way ANOVA (A, C, E, G) and followed by uncorrected Fisher's LSD post hoc test for phase comparisons (B, D, F, H). *p < 0.05, **p < 0.01, ***p < 0.001, ****p < 0.0001. All bar plots show mean ± SEM. Statistics found in Table S6.
**Figure S3:** Downscaling by random selection from each cohort shows similar results as in the main manuscript. Adolescent (p36) and adult (9w) mice were sleep‐disturbed (SD) or left undisrupted (Ctrl), social‐isolated (SI) or group‐housed (social). A tail suspension test (TST) was performed to elucidate despair‐like behaviour. (A) Total immobility time for all adolescent groups (*n* = 7–26) and young adult mice (*n* = 8). A three‐way ANOVA with repeated measures and Tukey multiple correction test, *p < 0.05. (B) TST immobility over the 6‐min test duration in the adolescent group (*n* = 7–26). (C) TST immobility over the six‐minute test duration in the young adult group (*n* = 8). A three‐way ANOVA with repeated measures, *p < 0.05. All data is plotted with mean ± standard error of the mean. Statistics found in Tables S7 and S8.
**Figure S4:** Downscaling by random selection from each cohort shows similar results as in the main manuscript. Adolescent (p36) and adult (9w) mice were sleep‐disturbed (SD) or left undisrupted (Ctrl), social‐isolated (SI) or group‐housed (social). An open field test (OFT) was performed to elucidate anxiety‐like behaviour. (A) Mean velocity, (B) mean distance moved and (C) anxiety‐like behaviour was measured for both adolescent (*n* = 7–26) and young adult (*n* = 8) mice. A three‐way ANOVA with repeated measures was used, **p < 0.01, ***p < 0.001, ****p < 0.0001. All data is plotted with mean ± standard error of the mean. Statistics Table S9.
**Table S1:** Two‐way ANOVA results for home‐cage activity in SD, SI, social and Ctrl mice, both adolescent and young adult (Figure 1).
**Table S2:** Three‐way ANOVA results of the total immobility in the tail suspension test (TST) (Figure 2).
**Table S3:** Three‐way ANOVA results of the immobility duration over time in the tail suspension test (TST) (Figure 2).
**Table S4:** Three‐way ANOVA results of the open field test (OFT) measures (Figure 3).
**Table S5:** One‐way ANOVA, Tukey multiple comparisions test—no difference between control groups (Figure S1).
**Table S6:** Downscaled—Two‐way ANOVA results for home‐cage activity in SD, SI, social and Ctrl mice, both adolescent and young adult (Figure S2). Statistics for adolescent isolation, adult socially housed and adult social isolation are presented in Table S1.
**Table S7:** Downscaled—Three‐way ANOVA results of the total immobility in the tail suspension test (TST) (Figure S3).
**Table S8:** Downscaled—Three‐way ANOVA results of the immobility duration over time in the tail suspension test (TST) (Figure S3). Statistics for adults are presented in Table S1.
**Table S9:** Downscaled—Three‐way ANOVA results of the open field test (OFT) measures (Figure S4).

## Data Availability

The data that support the findings of this study are available from the corresponding author upon reasonable request.
