# Supplementary material for: Synergistic Impact of Sleep Disturbance and Social Isolation in Adult but Not Adolescent Female Mice
Source: J Sleep Res. 2025 Sep 25;35(3):e70213. doi: 10.1111/jsr.70213 (PMC13193512; doi:10.1111/jsr.70213)
Supplement: Supplementary file 1 — Figure S1: No significant differences between adolescent controls. Controls of the repeated cohorts (first control against social isolation [SI], second control against sleep disturbance [SD] and third control against both SD + SI) were tested to ensure no difference between them. Statistics found in Table S5. Figure S2: Downscaling by random selection from each cohort shows similar results as in the main manuscript. Adolescent (p36) and adult (9 weeks) mice were either sleep‐disturbed (SD) or left undisturbed (Ctrl), and housed either socially (group‐housed) or in social isolation (SI). (A, C, E, G) Mean locomotion activity index over 6 days/h plotted from zeitgeber time (ZT) (A) socially housed adolescent mice (SD vs. Ctrl), 7 (n = 10). (B) Data from (A), separated early (ZT12‐17) and late dark phases (ZT18‐23) (n = 10). (C) Socially isolated adolescent mice (SD vs. Ctrl) (n = 6–10). (D) Data from (C), separated into early and late dark phases (n = 5–10). (E) Socially housed young adult mice (SD vs. Ctrl) (n = 8). (F) Data from (E), separated into early and late dark phases (n = 8). (G) Socially isolated young adult mice (SD vs. Ctrl) (n = 8). (H) Data from (G), separated into early and late dark phases (n = 8). Data analysed using repeated‐measures two‐way ANOVA (A, C, E, G) and followed by uncorrected Fisher's LSD post hoc test for phase comparisons (B, D, F, H). *p < 0.05, **p < 0.01, ***p < 0.001, ****p < 0.0001. All bar plots show mean ± SEM. Statistics found in Table S6. Figure S3: Downscaling by random selection from each cohort shows similar results as in the main manuscript. Adolescent (p36) and adult (9w) mice were sleep‐disturbed (SD) or left undisrupted (Ctrl), social‐isolated (SI) or group‐housed (social). A tail suspension test (TST) was performed to elucidate despair‐like behaviour. (A) Total immobility time for all adolescent groups (n = 7–26) and young adult mice (n = 8). A three‐way ANOVA with repeated measures and Tukey multiple correction test, [file JSR-35-e70213-s001.docx]

Supplementary materials


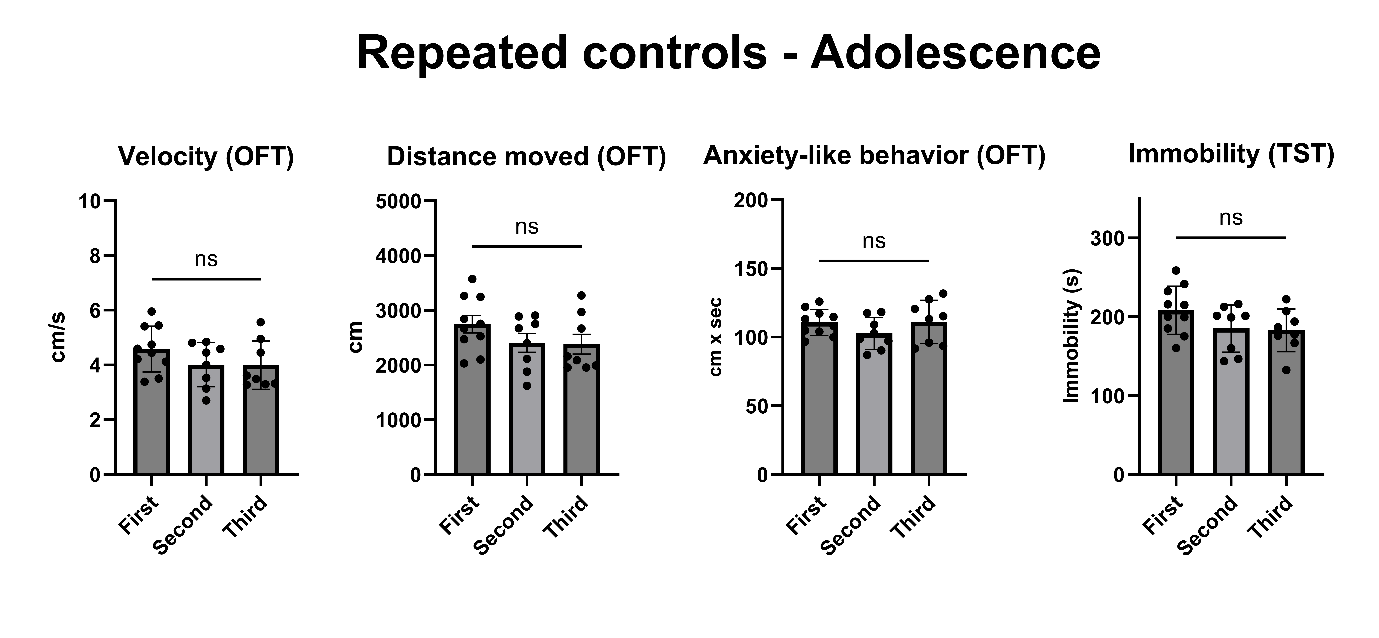
**Supplementary Figure 1: No significant differences between adolescent controls.** Controls of the repeated cohorts (first control against social isolation (SI), second control against sleep disturbance (SD), and third control against both SD + SI) were tested to ensure no difference between them. Statistics found in the Suppl. Table 5.


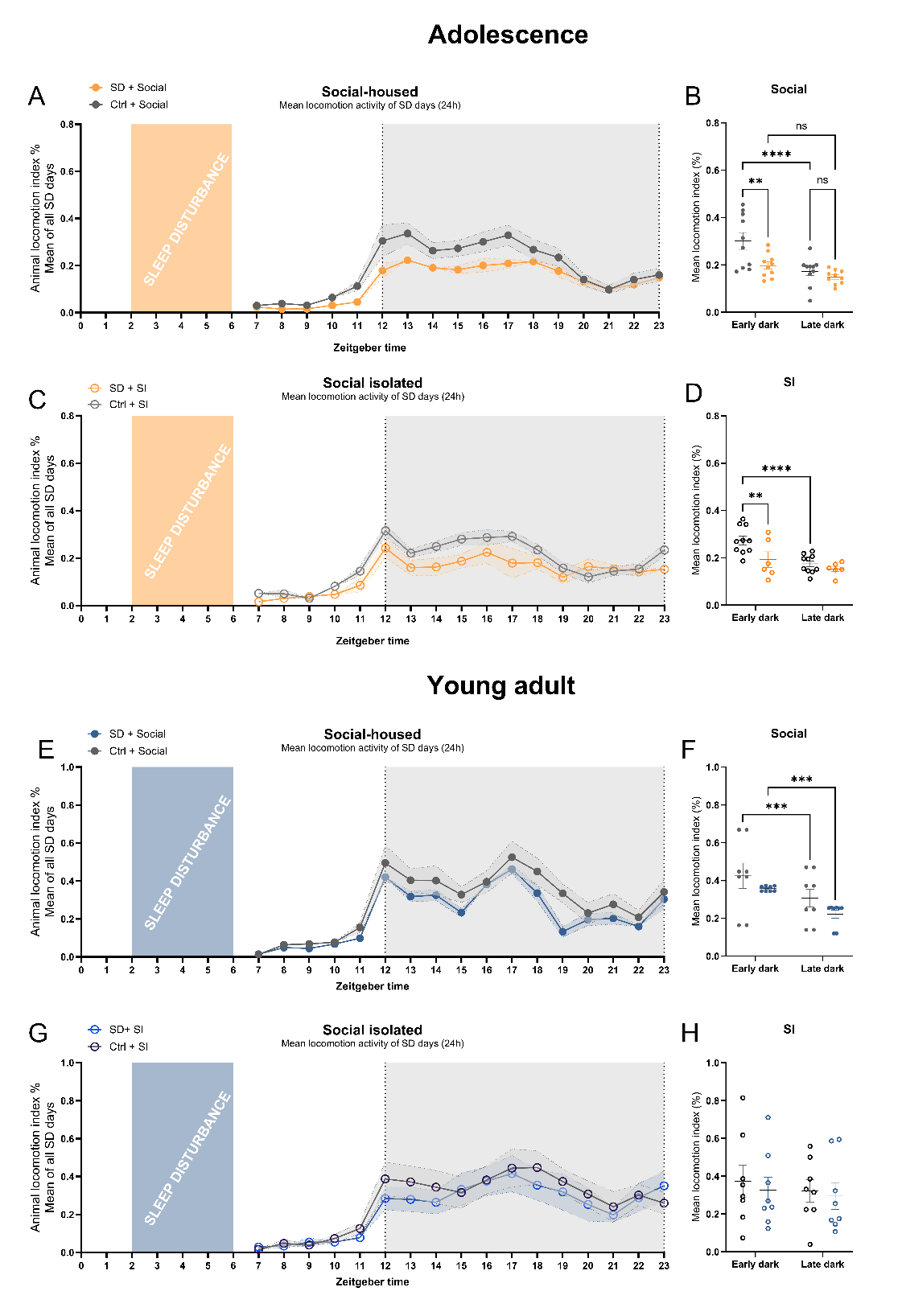


**Supplementary Figure 2: Downscaling by random selection from each cohort shows similar results as in the main manuscript.** Adolescent (P36) and adult (9 weeks) mice were either sleep-disturbed (SD) or left undisturbed (Ctrl), and housed either socially (group-housed) or in social isolation (SI). A,C,E, and G show mean locomotion activity index over six days per hour plotted from zeitgeber time (ZT) (A) socially housed adolescent mice (SD vs. Ctrl), 7 (n = 10). (B) Data from (A), separated early (ZT12-17) and late dark phases (ZT18-23) (n = 10). (C) Socially isolated adolescent mice (SD vs. Ctrl) (n = 6–10). (D) Data from (C), separated into early and late dark phases (n = 5–10). (E) Socially housed young adult mice (SD vs. Ctrl) (n = 8). (F) Data from (E), separated into early and late dark phases (n = 8). (G) Socially isolated young adult mice (SD vs. Ctrl) (n = 8). (H) Data from (G), separated into early and late dark phases (n = 8). Data analyzed using repeated-measures two-way ANOVA (A, C, E, G) and followed by uncorrected Fisher’s LSD post hoc test for phase comparisons (B, D, F, H). **p* < 0.05, **p < 0.01, ****p* < 0.001, ****p < 0.0001. All bar plots show mean ± SEM. Statistics found in the Suppl. Table 6.


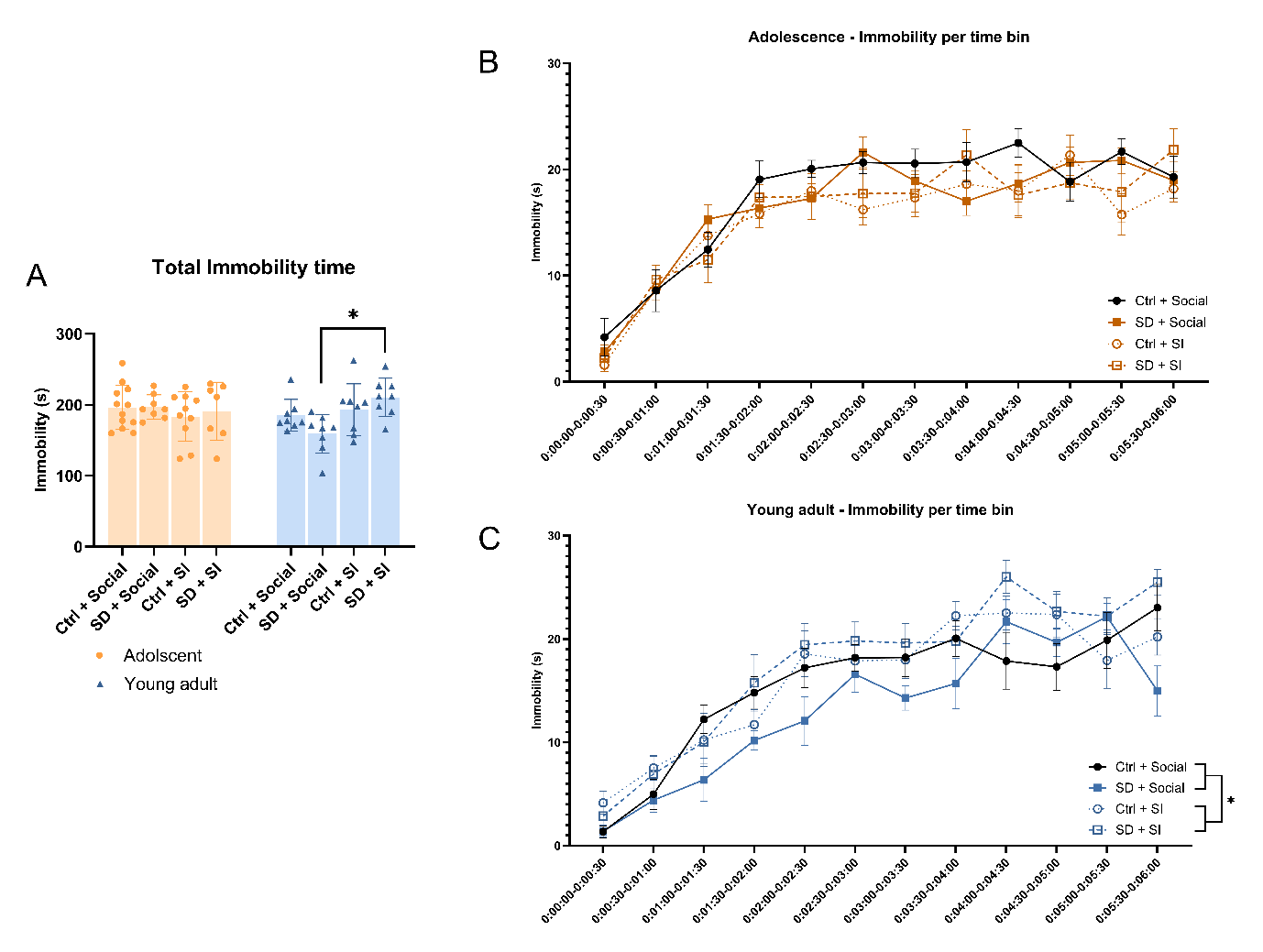


**Supplementary Figure 3: Downscaling by random selection from each cohort shows similar results as in the main manuscript.** Adolescent (p36) and adult (9w) mice were sleep-disturbed (SD) or left undisrupted (Ctrl), social-isolated (SI) or group-housed (social). A tail suspension test (TST) was performed to elucidate despair-like behavior. (A) Total immobility time for all adolescent groups (n =7-26) and young adult mice (n = 8). A three-way ANOVA with repeated measures and Tukey multiple correction test, *p < 0.05. (B) TST immobility over the 6-minute test duration in the adolescent group (n =7-26). (C) TST immobility over the six-minute test duration in the young adult group (n = 8). A three-way ANOVA with repeated measures, *p < 0.05. All data is plotted with mean ± standard error of the mean. Statistics found in Suppl. Tables 7 and 8.


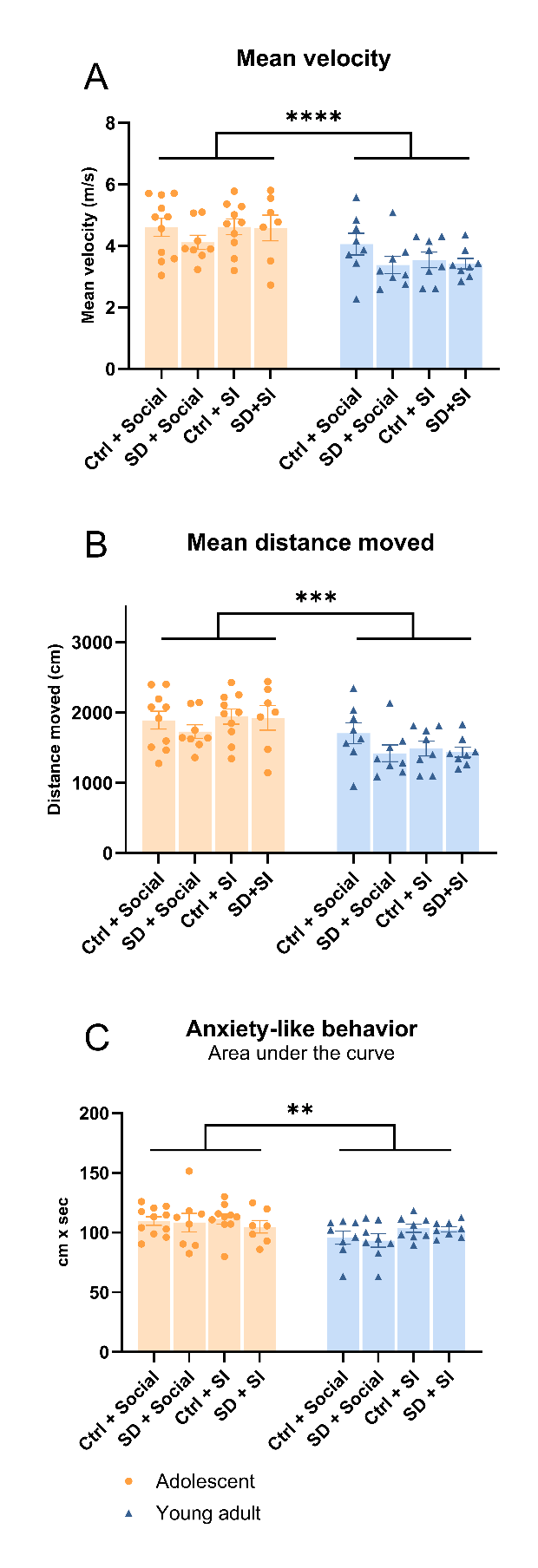


**Supplementary Figure 4: Downscaling by random selection from each cohort shows similar results as in the main manuscript.** Adolescent (p36) and adult (9w) mice were sleep-disturbed (SD) or left undisrupted (Ctrl), social-isolated (SI) or group-housed (social). An open field test (OFT) was performed to elucidate anxiety-like behavior. (A) Mean velocity, (B) mean distance moved, and (C) anxiety-like behavior was measured for both adolescent (n = 7-26) and young adult (n = 8) mice. A three-way ANOVA with repeated measures was used, **p < 0.01, ***p < 0.001, ****p < 0.0001. All data is plotted with mean ± standard error of the mean. Statistics Suppl. Table 9.

**Supplementary Table 1: Two-way ANOVA results for home-cage activity in SD, SI, social, and Ctrl mice, both adolescent and young adult (Figure 1).**

| Home-cage activity during SI and SD | | | | | | | | | | | | |
| --- | --- | --- | --- | --- | --- | --- | --- | --- | --- | --- | --- | --- |
| Adolescence – Social-Housed | | | | | | | | | | | | |
| Source of Variation | | | **F (DFn, DFd)** | | | | **P value** | | | | | **P value summary** |
| *SD x Time* | | | F (5.624, 461.2) = 5.562 | | | | <0.0001 | | | | | **** |
| *Time* | | | F (5.624, 461.2) = 113.5 | | | | <0.0001 | | | | | **** |
| *SD* | | | F (1, 82) = 24.25 | | | | <0.0001 | | | | | **** |
| *Subject* | | | F (82, 1394) = 8.272 | | | | <0.0001 | | | | | **** |
| Early vs. dark phase | | | | | | | | | | | | |
| Source of Variation | | | **F (DFn, DFd)** | | | | **P value** | | | | | **P value summary** |
| *SD x dark phase* | | | F (1, 18) = 5.288 | | | | 0.0258 | | | | | * |
| *Dark phase* | | | F (1, 18) = 26.20 | | | | <0.0001 | | | | | **** |
| *SD* | | | F (1, 18) = 5.994 | | | | <0.0001 | | | | | **** |
| *Subject* | | | F (18, 18) = 2.336 | | | | <0.0001 | | | | | **** |
| 2way ANOVA multiple comparison | | | | | | | | | | | | |
| Early dark | | | | | | **Late dark** | | | | | | |
| Group | **P value** | | | **P value summary** | | **Group** | | | **P value** | **P value summary** | | |
| Ctrl vs SD | <0.0001 | | | ******** | | Ctrl vs SD | | | 0.0039 | ** | | |
| Ctrl | | | | | | **SD** | | | | | | |
| Group | **P value** | | | **P value summary** | | **Group** | **P value** | | | **P value summary** | | |
| Early vs Late | <0.0001 | | | ******** | | Early vs Late | <0.0001 | | | ******** | | |
| Adolescence – Social isolation | | | | | | | | | | | | |
| Source of Variation | | | **F (DFn, DFd)** | | | | **P value** | | | | | **P value summary** |
| *SD x Time* | | | F (5.707, 79.90) = 1.582 | | | | 0.1664 | | | | | ns |
| *Time* | | | F (5.707, 79.90) = 24.44 | | | | <0.0001 | | | | | **** |
| *SD* | | | F (1, 14) = 6.046 | | | | 0.0276 | | | | | * |
| *Subject* | | | F (14, 238) = 6.113 | | | | <0.0001 | | | | | **** |
| Early vs. dark phase | | | | | | | | | | | | |
| Source of Variation | | | **F (DFn, DFd)** | | | | **P value** | | | | | **P value summary** |
| *SD x dark phase* | | | F (1, 14) = 4.724 | | | | 0.0474 | | | | | * |
| *Dark phase* | | | F (1, 14) = 25.90 | | | | 0.0002 | | | | | *** |
| *SD* | | | F (1, 14) = 4.560 | | | | 0.0509 | | | | | ns |
| *Subject* | | | F (14, 14) = 3.141 | | | | 0.0201 | | | | | * |
| 2way ANOVA multiple comparison | | | | | | | | | | | | |
| Early dark | | | | | | **Late dark** | | | | | | |
| Group | **P value** | | | **P value summary** | | **Group** | **P value** | | | **P value summary** | | |
| Ctrl vs SD | 0.0067 | | | ****** | | Ctrl vs SD | 0.4353 | | | ns | | |
| Ctrl | | | | | | **SD** | | | | | | |
| Group | **P value** | | | **P value summary** | | **Group** | **P value** | | | **P value summary** | | |
| Early vs Late | <0.0001 | | | ******** | | Early vs Late | 0.0865 | | | ns | | |
| Adult – Social-Housed | | | | | | | | | | | | |
| Source of Variation | | | **F (DFn, DFd)** | | | | **P value** | | | | | **P value summary** |
| *SD x Time* | | | F (3.433, 48.06) = 1.159 | | | | 0.3381 | | | | | ns |
| *Time* | | | F (3.433, 48.06) = 43.42 | | | | <0.0001 | | | | | **** |
| *SD* | | | F (1, 14) = 2.167 | | | | 0.1631 | | | | | ns |
| *Subject* | | | F (14, 238) = 15.40 | | | | <0.0001 | | | | | **** |
| Early vs. dark phase | | | | | | | | | | | | |
| Source of Variation | | | **F (DFn, DFd)** | | | | **P value** | | | | | **P value summary** |
| *SD x dark phase* | | | F (1, 14) = 0.2223 | | | | 0.6446 | | | | | ns |
| *Dark phase* | | | F (1, 14) = 46.14 | | | | <0.0001 | | | | | **** |
| *SD* | | | F (1, 14) = 1.771 | | | | 0.2046 | | | | | ns |
| *Subject* | | | F (14, 14) = 9.572 | | | | <0.0001 | | | | | ns |
| 2way ANOVA multiple comparison | | | | | | | | | | | | |
| Early dark | | | | | | **Late dark** | | | | | | |
| Group | | **P value** | | | **P value summary** | **Group** | | **P value** | | | **P value summary** | |
| Ctrl vs SD | | 0.2717 | | | ns | Ctrl vs SD | | 0.1692 | | | ns | |
| Ctrl | | | | | | **SD** | | | | | | |
| Group | | **P value** | | | **P value summary** | **Group** | | **P value** | | | **P value summary** | |
| Early vs Late | | 0.0005 | | | ******* | Early vs Late | | 0.0002 | | | ** | |
| Adult – Social isolation | | | | | | | | | | | | |
| Source of Variation | | | **F (DFn, DFd)** | | | | **P value** | | | | | **P value summary** |
| *SD x Time* | | | F (3.071, 42.99) = 0.6416 | | | | 0.5960 | | | | | ns |
| *Time* | | | F (3.071, 42.99) = 21.14 | | | | <0.0001 | | | | | **** |
| *SD* | | | F (1, 14) = 0.1716 | | | | 0.6849 | | | | | ns |
| *Subject* | | | F (14, 238) = 23.88 | | | | <0.0001 | | | | | **** |
| Early vs. dark phase | | | | | | | | | | | | |
| Source of Variation | | | **F (DFn, DFd)** | | | | **P value** | | | | | **P value summary** |
| *SD x dark phase* | | | F (1, 14) = 0.1380 | | | | 0.7158 | | | | | ns |
| *Dark phase* | | | F (1, 14) = 2.165 | | | | 0.1633 | | | | | ns |
| *SD* | | | F (1, 14) = 0.1587 | | | | 0.6964 | | | | | ns |
| *Subject* | | | F (14, 14) = 11.47 | | | | <0.0001 | | | | | **** |
| 2way ANOVA multiple comparison | | | | | | | | | | | | |
| Early dark | | | | | | **Late dark** | | | | | | |
| Group | | **P value** | | | **P value summary** | **Group** | | **P value** | | | **P value summary** | |
| Ctrl vs SD | | 0.6299 | | | ns | Ctrl vs SD | | 0.7839 | | | ns | |
| Ctrl | | | | | | **SD** | | | | | | |
| Group | | **P value** | | | **P value summary** | **Group** | | **P value** | | | **P value summary** | |
| Early vs Late | | 0.2135 | | | ns | Early vs Late | | 0.4496 | | | ns | |

**Supplementary Table 2: Three-way ANOVA results of the total immobility in the tail suspension test (TST) (Figure 2).**

Abbreviations: Social – group-housed, SI – social isolation, ctrl – control, SD – sleep disturbance

| Total immobility in TST | | | | | | | |  |
| --- | --- | --- | --- | --- | --- | --- | --- | --- |
| Source of Variation | | **F (DFn, DFd)** | | **P value** | | **P value summary** | |  |
| *Age* | | F (1, 75) = 0.3193 | | 0.5737 | | ns | |  |
| *Social vs. SI* | | F (1, 75) = 2.288 | | 0.1346 | | ns | |  |
| *Ctrl vs. SD* | | F (1, 75) = 0.01025 | | 0.9196 | | ns | |  |
| *Age x Social vs. SI* | | F (1, 75) = 6.698 | | 0.0116 | | * | |  |
| *Age x Ctrl vs. SD* | | F (1, 75) = 0.4645 | | 0.4976 | | ns | |  |
| *Social vs. SI x Ctrl vs. SD* | | F (1, 75) = 2.622 | | 0.1096 | | ns | |  |
| *Age x Social vs. SI x Ctrl vs. SD* | | F (1, 75) = 1.811 | | 0.1824 | | ns | |  |
| 2way multiple comparisons | | | | | | | |  |
| Adolescence | | | Adult | | | | |  |
| Group | P value | Summary | Group | | P value | | Summary | |
| Ctrl + Social vs. SD + Social | 0.9906 | ns | Ctrl + Social vs. SD + Social | | 0.3374 | | ns | |
| Ctrl + Social vs. Ctrl + SI | 0.8240 | ns | Ctrl + Social vs. Ctrl + SI | | 0.9488 | | ns | |
| Ctrl + Social vs. SD + SI | 0.9984 | ns | Ctrl + Social vs. SD + SI | | 0.3437 | | ns | |
| SD + Social vs. Ctrl + SI | 0.7876 | ns | SD + Social vs. Ctrl + SI | | 0.1250 | | ns | |
| SD + Social vs. SD + SI | 0.9827 | ns | SD + Social vs. SD + SI | | 0.0065 | | ** | |
| Ctrl + SI vs. SD + SI | 0.9564 | ns | Ctrl + SI vs. SD + SI | | 0.6691 | | ns | |

**Supplementary Table 3: Three-way ANOVA results of the immobility duration over time in the tail suspension test (TST) (Figure 2).**

Abbreviations: Social – group-housed, SI – social isolation, ctrl – control, SD – sleep disturbance

| Immobility in TST over time | | | |
| --- | --- | --- | --- |
| Adolescent | | | |
| Source of Variation | **F (DFn, DFd)** | **P value** | **P value summary** |
| *Time* | F (11, 513) = 58.23 | <0.0001 | **** |
| *Social vs. SI* | F (1, 47) = 0.6163 | 0.4364 | ns |
| *Ctrl vs SD* | F (1, 47) = 0.3614 | 0.5506 | ns |
| *Time x Social vs SI* | F (11, 513) = 1.776 | 0.0553 | ns |
| *Time x Ctrl vs SD* | F (11, 513) = 0.5775 | 0.8476 | ns |
| *Social vs SI x Ctrl vs SD* | F (1, 47) = 0.03218 | 0.8584 | ns |
| *Time x Social vs. SI x Ctrl vs. SD* | F (11, 513) = 1.198 | 0.285 | ns |
| Adult | | | |
| Source of Variation | **F (DFn, DFd)** | **P value** | **P value summary** |
| *Time* | F (7.148, 200.2) = 57.17 | <0.0001 | **** |
| *Social vs. SI* | F (1, 28) = 8.558 | 0.0067 | ** |
| *Ctrl vs SD* | F (1, 28) = 0.1713 | 0.6821 | ns |
| *Time x Social vs. SI* | F (11, 308) = 0.9289 | 0.5127 | ns |
| *Time x Ctrl vs. SD* | F (11, 308) = 1.704 | 0.0716 | ns |
| *Social vs. SI x Ctrl vs. SD* | F (1, 28) = 4.478 | 0.0434 | * |
| *Time x Social vs. SI x Ctrl vs. SD* | F (11, 308) = 1.782 | 0.0563 | ns |

**Supplementary Table 4: Three-way ANOVA results of the open field test (OFT) measures (Figure 3).**

Abbreviations: Social – group-housed, SI – social isolation, ctrl – control, SD – sleep disturbance

| Measures of OFT | | | |
| --- | --- | --- | --- |
| Velocity | | | |
| Source of Variation | **F (DFn, DFd)** | **P value** | **P value summary** |
| *Age* | F (1, 75) = 17.74 | <0.0001 | **** |
| *Social vs. SI* | F (1, 75) = 0.01085 | 0.9173 | ns |
| *Ctrl vs SD* | F (1, 75) = 2.351 | 0.1294 | ns |
| *Age x Social vs SI* | F (1, 75) = 1.571 | 0.2139 | ns |
| *Age x Ctrl vs SD* | F (1, 75) = 0.1796 | 0.6729 | ns |
| *Social vs SI x Ctrl vs SD* | F (1, 75) = 1.289 | 0.2599 | ns |
| *Age x Social vs SI x Ctrl vs SD* | F (1, 75) = 0.05019 | 0.8233 | ns |
| Distance travelled | | | |
| Source of Variation | **F (DFn, DFd)** | **P value** | **P value summary** |
| *Age* | F (1, 75) = 17.73 | <0.0001 | **** |
| *Social vs. SI* | F (1, 75) = 0.01107 | 0.9165 | ns |
| *Ctrl vs SD* | F (1, 75) = 2.354 | 0.1292 | ns |
| *Age x Social vs SI* | F (1, 75) = 1.574 | 0.2135 | ns |
| *Age x Ctrl vs SD* | F (1, 75) = 0.1789 | 0.6736 | ns |
| *Social vs SI x Ctrl vs SD* | F (1, 75) = 1.291 | 0.2595 | ns |
| *Age x Social vs SI x Ctrl vs SD* | F (1, 75) = 0.04963 | 0.8243 | ns |
| Anxiety-like Behavior | | | |
| Source of Variation | **F (DFn, DFd)** | **P value** | **P value summary** |
| *Age* | F (1, 75) = 8.238 | 0.0053 | ** |
| *Social vs. SI* | F (1, 75) = 1.598 | 0.2100 | ns |
| *Ctrl vs SD* | F (1, 75) = 0.5854 | 0.4466 | ns |
| *Age x Social vs SI* | F (1, 75) = 1.810 | 0.1826 | ns |
| *Age x Ctrl vs SD* | F (1, 75) = 0.06394 | 0.8011 | ns |
| *Social vs SI x Ctrl vs SD* | F (1, 75) = 0.1557 | 0.6943 | ns |
| *Age x Social vs SI x Ctrl vs SD* | F (1, 75) = 0.3876 | 0.5355 | ns |

**Supplementary Table 5: One-way ANOVA, Tukey multiple comparisions test - No difference between control groups (Suppl. Figure 1)**

| Behavioral test - Control | | | | |
| --- | --- | --- | --- | --- |
| Velocity | | | | |
| Source of Variation | | **P value** | | **P value summary** |
| First vs. Second | | 0.3358 | | ns |
| First vs. Third | | 0.2929 | | ns |
| Second vs. Third | | 0.9957 | | ns |
| Distance travelled | | | | |
| Source of Variation | | **P value** | | **P value summary** |
| First vs. Second | | 0.3418 | | ns |
| First vs. Third | | 0.3247 | | ns |
| Second vs. Third | | 0.9994 | | ns |
| Anxiety-like behavior | | | | |
| Source of Variation | | **P value** | | **P value summary** |
| First vs. Second | | 0.3726 | | ns |
| First vs. Third | | 0.9953 | | ns |
| Second vs. Third | | 0.3639 | | ns |
| Immobility | | | | |
| Source of Variation | **P value** | | **P value summary** | |
| First vs. Second | 0.2370 | | ns | |
| First vs. Third | 0.1804 | | ns | |
| Second vs. Third | 0.9868 | | ns | |

**Supplementary Table 6: Downscaled - Two-way ANOVA results for home-cage activity in SD, SI, social, and Ctrl mice, both adolescent and young adult (Suppl. Figure 2).** Statistics for adolescent isolation, adult socially housed, and adult social isolation are presented in Supplementary Table 1.

| Home-cage activity during SI and SD | | | | | | | | | |  |
| --- | --- | --- | --- | --- | --- | --- | --- | --- | --- | --- |
| Adolescence – Social-Housed | | | | | | | | | |  |
| Source of Variation | | **F (DFn, DFd)** | | | | **P value** | | | **P value summary** |  |
| *SD x Time* | | F (4.208, 75.74) = 1.559 | | | | 0.1913 | | | ns |  |
| *Time* | | F (4.208, 75.74) = 27.89 | | | | <0.0001 | | | **** |  |
| *SD* | | F (1, 18) = 7.816 | | | | 0.0119 | | | * |  |
| *Subject* | | F (18, 306) = 5.520 | | | | <0.0001 | | | **** |  |
| Early vs. dark phase | | | | | | | | | |  |
| Source of Variation | | **F (DFn, DFd)** | | | | **P value** | | | **P value summary** |  |
| *SD x dark phase* | | F (1, 18) = 5.288 | | | | 0.0337 | | | * |  |
| *Dark phase* | | F (1, 18) = 26.20 | | | | <0.0001 | | | **** |  |
| *SD* | | F (1, 18) = 5.994 | | | | 0.0248 | | | * |  |
| *Subject* | | F (18, 18) = 2.336 | | | | 0.0400 | | | * |  |
| 2way ANOVA multiple comparison | | | | | | | | | | |
| Early dark | | | | | **Late dark** | | | | | |
| Group | | **P value** | | **P value summary** | **Group** | | | **P value** | **P value summary** | |
| Ctrl vs SD | | 0.0021 | | ****** | Ctrl vs SD | | | 0.4348 | ns | |
| Ctrl | | | | | **SD** | | | | | |
| Group | | **P value** | | **P value summary** | **Group** | **P value** | | | **P value summary** | |
| Early vs Late | | <0.0001 | | ******** | Early vs Late | 0.0616 | | | ns | |

**Supplementary Table 7: Downscaled - Three-way ANOVA results of the total immobility in the tail suspension test (TST) (Suppl. Figure 3).**

Abbreviations: Social – group-housed, SI – social isolation, ctrl – control, SD – sleep disturbance

| Total immobility in TST | | | | | | | | |
| --- | --- | --- | --- | --- | --- | --- | --- | --- |
| Source of Variation | | **F (DFn, DFd)** | | | **P value** | **P value summary** | | |
| Age | | F (1, 61) = 0.5690 | | | 0.4536 | ns | | |
| Social vs SI | | F (1, 61) = 1.663 | | | 0.2021 | ns | | |
| Ctrl vs SD | | F (1, 61) = 0.009784 | | | 0.9215 | ns | | |
| Age x Social vs SI | | F (1, 61) = 7.531 | | | 0.0080 | ** | | |
| Age x Ctrl vs SD | | F (1, 61) = 0.2227 | | | 0.6387 | ns | | |
| Social vs SI x Ctrl vs SD | | F (1, 61) = 3.209 | | | 0.0782 | ns | | |
| Age x Social vs SI x Ctrl vs SD | | F (1, 61) = 1.267 | | | 0.2648 | ns | | |
|  | |  | | |  |  | | |
| 2way multiple comparisons | | | | | | | | |
| Adolescence | | | | Adult | | | | |
| Group | P value | | Summary | Group | | | P value | Summary |
| Ctrl + Social vs. SD + Social | >0.9999 | | ns | Ctrl + Social vs. SD + Social | | | 0.6867 | ns |
| Ctrl + Social vs. Ctrl + SI | 0.9266 | | ns | Ctrl + Social vs. Ctrl + SI | | | 0.9993 | ns |
| Ctrl + Social vs. SD + SI | 0.9993 | | ns | Ctrl + Social vs. SD + SI | | | 0.6944 | ns |
| SD + Social vs. Ctrl + SI | 0.9808 | | ns | SD + Social vs. Ctrl + SI | | | 0.3404 | ns |
| SD + Social vs. SD + SI | >0.9999 | | ns | SD + Social vs. SD + SI | | | 0.0255 | * |
| Ctrl + SI vs. SD + SI | 0.9995 | | ns | Ctrl + SI vs. SD + SI | | | 0.9441 | ns |

**Supplementary Table 8: Downscaled - Three-way ANOVA results of the immobility duration over time in the tail suspension test (TST) (Suppl. Figure 3).** Statistics for adults are presented in Supplementary Table 1.

Abbreviations: Social – group-housed, SI – social isolation, ctrl – control, SD – sleep disturbance

| Immobility in TST over time | | | |
| --- | --- | --- | --- |
| Adolescent | | | |
| Source of Variation | **F (DFn, DFd)** | **P value** | **P value summary** |
| *Time* | F (11, 348) = 53.70 | <0.0001 | **** |
| *Social vs. SI* | F (1, 32) = 2.394 | 0.1317 | ns |
| *Ctrl vs SD* | F (1, 32) = 0.05357 | 0.8184 | ns |
| *Time x Social vs SI* | F (11, 348) = 1.544 | 0.1142 | ns |
| *Time x Ctrl vs SD* | F (11, 348) = 0.6755 | 0.7619 | ns |
| *Social vs SI x Ctrl vs SD* | F (1, 32) = 0.9453 | 0.3382 | ns |
| *Time x Social vs. SI x Ctrl vs. SD* | F (11, 348) = 1.269 | 0.2404 | ns |

**Supplementary Table 9: Downscaled - Three-way ANOVA results of the open field test (OFT) measures (Suppl. Figure 4).**

Abbreviations: Social – group-housed, SI – social isolation, ctrl – control, SD – sleep disturbance

| Measures of OFT | | | |
| --- | --- | --- | --- |
| Velocity | | | |
| Source of Variation | **F (DFn, DFd)** | **P value** | **P value summary** |
| *Age* | F (1, 60) = 18.32 | <0.0001 | **** |
| *Social vs. SI* | F (1, 60) = 0.0001183 | 0.9914 | ns |
| *Ctrl vs SD* | F (1, 60) = 2.615 | 0.1111 | ns |
| *Age x Social vs SI* | F (1, 60) = 1.331 | 0.2532 | ns |
| *Age x Ctrl vs SD* | F (1, 60) = 0.1080 | 0.7435 | ns |
| *Social vs SI x Ctrl vs SD* | F (1, 60) = 1.492 | 0.2266 | ns |
| *Age x Social vs SI x Ctrl vs SD* | F (1, 60) = 0.01691 | 0.8970 | ns |
| Distance travelled | | | |
| Source of Variation | **F (DFn, DFd)** | **P value** | **P value summary** |
| *Age* | F (1, 60) = 18.31 | <0.0001 | **** |
| *Social vs. SI* | F (1, 60) = 0.0001392 | 0.9906 | ns |
| *Ctrl vs SD* | F (1, 60) = 2.619 | 0.1109 | ns |
| *Age x Social vs SI* | F (1, 60) = 1.333 | 0.2528 | ns |
| *Age x Ctrl vs SD* | F (1, 60) = 0.1074 | 0.7443 | ns |
| *Social vs SI x Ctrl vs SD* | F (1, 60) = 1.495 | 0.2262 | ns |
| *Age x Social vs SI x Ctrl vs SD* | F (1, 60) = 0.01656 | 0.8980 | ns |
| Anxiety-like Behavior | | | |
| Source of Variation | **F (DFn, DFd)** | **P value** | **P value summary** |
| *Age* | F (1, 60) = 8.048 | 0.0062 | ** |
| *Social vs. SI* | F (1, 60) = 1.246 | 0.2689 | ns |
| *Ctrl vs SD* | F (1, 60) = 0.6793 | 0.4131 | ns |
| *Age x Social vs SI* | F (1, 60) = 1.901 | 0.1731 | ns |
| *Age x Ctrl vs SD* | F (1, 60) = 0.1119 | 0.7392 | ns |
| *Social vs SI x Ctrl vs SD* | F (1, 60) = 0.08094 | 0.7770 | ns |
| *Age x Social vs SI x Ctrl vs SD* | F (1, 60) = 0.2525 | 0.6172 | ns |
